# Nationwide seroprevalence of SARS-CoV-2 Delta variant and five Omicron sublineages in companion cats and dogs in the USA: insights into their role in COVID-19 epidemiology

**DOI:** 10.1080/22221751.2024.2437246

**Published:** 2024-12-05

**Authors:** Subarna Barua, Nneka Vivian Iduu, Daniel Felipe Barrantes Murillo, Asfiha Tarannum, Hill Dimino, Suchita Barua, Yue Shu, Calvin Johnson, Megan R. Miller, Kelly Chenoweth, Peter Christopherson, Laura Huber, Theresa Wood, Kelley Turner, Chengming Wang

**Affiliations:** a Department of Pathobiology, College of Veterinary Medicine, Auburn University, Auburn, AL, USA; b College of Sciences and Mathematics, Auburn University, Auburn, AL, USA; c Center for Veterinary Medicine, U.S. Food and Drug Administration, Laurel, MD, USA

**Keywords:** SARS-CoV-2, Delta variant, Omicron sublineages, surrogate virus neutralization test, cats and dogs, USA

## Abstract

Understanding SARS-CoV-2 epidemiology in companion animals is critical for evaluating their role in viral transmission and their potential as sentinels for human infections. This large-scale serosurvey analyzed serum samples from 706 cats and 2,396 dogs collected across the USA in 2023 using a surrogate virus neutralization test (sVNT) to detect SARS-CoV-2 antibodies. Overall, 5.7% of cats and 4.7% of dogs tested positive for antibodies, with younger animals (under 12 months) showing significantly lower seropositivity rates (*p *= 0.0048). Additionally, we analyzed 153 positive samples for variant-specific antibody responses using six sVNT kits targeting the Delta variant and five Omicron sublineages. Among cats, 67.5% showed antibodies to Delta, with positivity rates for Omicron sublineages as follows: BA.1 (62.5%), BA.2 (42.5%), BA.4/BA.5 (77.5%), XBB (52.5%), and XBB.1.5 (45.0%). In dogs, 55.8% were positive for Delta, and Omicron sublineage rates were BA.1 (46.0%), BA.4/BA.5 (37.2%), XBB (58.4%), BA.2 (13.3%), and XBB.1.5 (9.7%). Given the close contact between companion animals and humans, and the persistence of antibodies against various SARS-CoV-2 variants and sublineages, our findings suggest that seroprevalence in cats and dogs may serve as valuable tool for tracking COVID-19 epidemiology.

## Introduction

Since its emergence in December 2019, Severe Acute Respiratory Syndrome Coronavirus 2 (SARS-CoV-2) has evolved significantly, developing multiple strains and sublineages [1 2]. These variants have demonstrated enhanced transmissibility, greater severity, and increased ability to evade immune responses, thereby shaping the course of the pandemic [3].

The original Wuhan strain, along with the D614G mutation that became dominant in early 2020, was soon followed by the highly transmissible Delta variant (B.1.617.2) in late 2020, associated with severe disease outcomes [4] (Table 1). In November 2021, the Omicron variant (B.1.1.529) had emerged, bringing forth several sublineages, including BA.1, BA.2, BA.4/BA.5, XBB, and XBB.1.5 [3]. BA.1 and BA.2 spread rapidly across the globe in early 2022, with BA.2 being particularly transmissible [5,6]. By mid-2022, BA.4/BA.5 emerged, demonstrating enhanced immune escape [7]. Later in 2022, XBB, a recombinant sublineage, and its offshoot XBB.1.5, were noted for their strong binding to the ACE2 receptor, which posed new challenges due to their high transmissibility and immune evasion [8] (Table 1). These evolving variants underscored the critical need for continuous surveillance and adaptive public health strategies.

**Table 1.** The seroprevalence of SARS-CoV-2 Delta and five Omicron sublineages in this study.

Variants	First reported			Seropositivity in cats	Seropositivity in dogs
	Global-human	USA-human	This study-animal		
Delta	October 2020, India [9]	March 2021, OK [9]	February 2023	27/40, 67.50%	63/113, 55.75%
Omicron, BA.1	November 2021, South Africa [10]	December 2021, CA [11]	January 2023	25/40, 62.50%	52/113, 46.02%
Omicron, BA.2	August 2022, India [12]	September 2022,	January 2023	21/40, 52.50%	66/113, 58.41%
Omicron, BA.4/BA.5	December 2021 and January 2022, South Africa [13]	April 2022, PA [14]	January 2023	31/40, 77.50%	42/113, 37.17%
Omicron, XBB	November 2021, South Africa [5]	January 2022, Arizona [15]	January 2023	17/40, 42.50%	15/113, 13.27%
Omicron, XBB.1.5	October 2022, NY [16]	October 2022, NY [16]	January 2023	18/40, 45.00%	11/113, 9.73%

While the global COVID-19 pandemic has significantly impacted human populations, its implications extend beyond human health to include other animals, particularly companion cats and dogs [17]. Initial reports of SARS-CoV-2 infections in cats and dogs [18,19] led to further studies demonstrating that these species are susceptible to SARS-CoV-2 infection, with cats showing greater susceptibility and the potential to transmit the virus to others [20,21]. This published evidence suggests that cats and dogs can acquire the virus and mount a sustained immune response, producing antibodies to multiple SARS-CoV-2 variants.

Despite these findings, the role of these animals in viral circulation and variant detection within human populations remains unclear. Given the close interactions between pets and humans, investigating SARS-CoV-2 prevalence in companion animals could yield important insights into viral transmission dynamics [22].

To address these knowledge gaps and explore the potential of companion animals as sentinels for SARS-CoV-2 surveillance, we conducted a comprehensive serosurvey using a surrogate virus neutralization test (sVNT) on a large cohort of cats and dogs across the United States. We aimed to determine the prevalence of antibodies against SARS-CoV-2 variants, including the Delta variant and five Omicron sublineages. The findings from this study offer updated insights into the epidemiology of SARS-CoV-2 in companion animals, highlighting significant risk factors such as age, and suggesting a potential role for these animals as sentinels for COVID-19.

## Materials and methods

### Feline and canine sera

**Figure 1.**
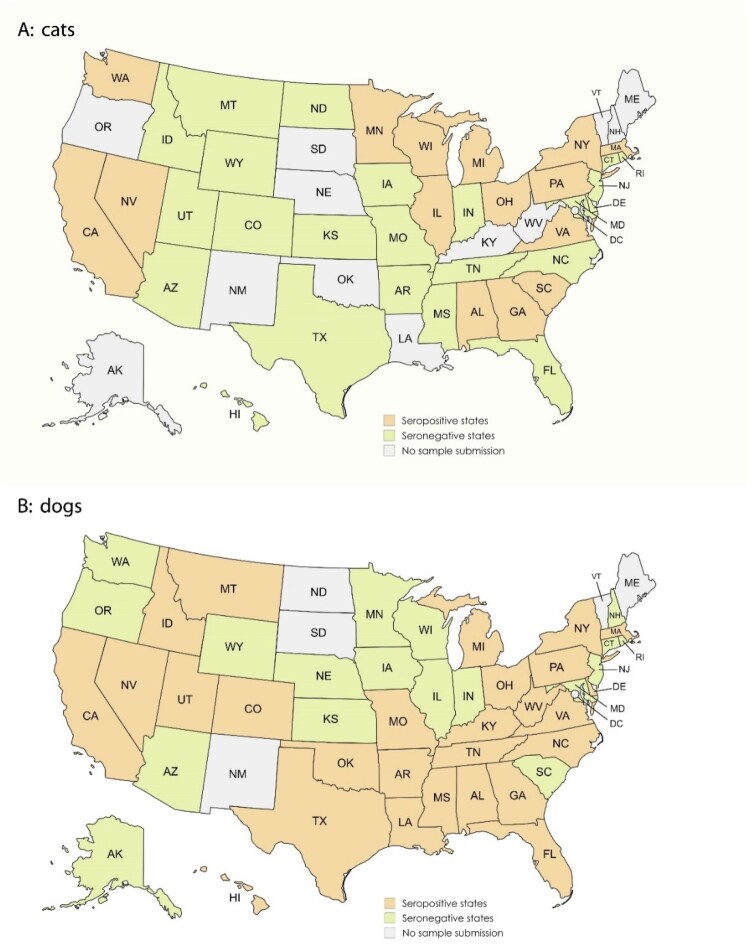
Geographical distribution of anti-SARS-CoV-2 seropositive samples in the continental United States. Feline and canine samples were collected from 49 states, of which 35 showed seropositivity while 14 states, with small sample sizes ranging from 3 to 29, did not have any samples that tested positive for SARS-CoV-2 antibodies. Antibody-positive samples were found in dogs from 15 out of 39 states (**A**), and in dogs from 27 of 46 states (B).

The study analyzed convenience serum samples from apparently healthy animals, including 706 cats (39 states) and 2,396 dogs (46 states) in the USA (Figure 1). These samples were submitted to the Virology & Serology Laboratory at Auburn University College of Veterinary Medicine between January and December 2023 for rabies titre testing which is a requirement for export for pet travel. Information regarding the COVID-19 status of the households from which these animals were sourced was unavailable. In addition, convenience serum samples from cattle (n = 64), horses (n = 80), goats (n = 41), and pigs (n = 15) submitted to the Clinical Pathology Laboratory at Auburn University College of Veterinary Medicine were also included in this study. All samples had been heat-inactivated at 56°C for 30 minutes for rabies testing.

### SARS-CoV-2 surrogate virus neutralization test

The SARS-CoV-2 Surrogate Virus Neutralization Test (sVNT) Kit was purchased from GenScript (New Jersey, USA) and used according to the manufacturer’s guidelines. This test is referred to as the standard sVNT for detecting antibodies against all SARS-CoV-2 strains.

Additionally, variant-specific SARS-CoV-2 sVNT kits from GenScript were utilized to detect antibodies against the Delta variant (B.1.617.2) and five Omicron (B.1.1.529) sublineages: BA.1, BA.2, BA.4/BA.5, XBB and XBB1.5 (Table 1). The variant-specific sVNT, which employs variant-specific RBD proteins, effectively identifies neutralizing antibody profiles unique to the Delta variant and five Omicron sublineages.

The variant-specific sVNT test was performed using standard ELISA kit reagents, as outlined in the manufacturer's manual, except for replacing the standard conjugate with variant-specific conjugates. Additionally, a SARS-CoV-2 Omicron standard was used for the variant sVNT test. The optical densities of the reactions were measured at a wavelength of 450 nm for each sample, as well as the positive and negative controls using a microplate reader SpectraMax® i Series-Spectramax Id3. Percentage inhibitions were then calculated using the following formula:

$$\begin{array}{rl} & \mathrm{Percent}\;\mathrm{Inhibition}\\ & =(1-\mathrm{sample}\;O.D.\;\mathrm{value}\\ & /\mathrm{negative}\;\mathrm{control}\;O.D.\;\mathrm{value})\times 100 \end{array}$$

Serum samples with percent inhibition values of ≥30% were classified as positive, whereas those with lower values were considered negative [23,24].

### Statistical analysis

All data were analyzed using the STATISTICA 7.1 software (Statsoft, Tulsa, Oklahoma). Chi-square tests were employed to analyze the significance of the relationships between sex, age, breed, and the presence of seroprevalence of SARS-CoV-2. Violin plots were generated on GraphPad Prism v10.3.0 (461) (GraphPad Software Inc., Boston, MA, USA). A web-based Venn diagram platform – Bioinformatics & Evolutionary Genomics (https://bioinformatics.psb.ugent.be/webtools/Venn/) was used to generate the Venn diagram.

## Results

### No significant difference in seroprevalence between feline and canine samples

In this study, the standard sVNT detected antibodies against SARS-CoV-2 in 4.9% of the submitted samples (153/3,102). Samples were collected from 49 states, of which 35 showed seropositivity (Figure 1). In contrast, 14 states, which had relatively small sample sizes ranging from 3 to 29 (mean: 9.14 ± 6.88), did not have any samples that tested positive for SARS-CoV-2 antibodies. Antibody-positive samples were found in dogs from 15 out of 39 states (Figure 1A), and in dogs from 27 of 46 states (Figure 1B). Antibody positivity rate did not differ significantly between cats (40/706, 5.7%, 15 states) and dogs (4.7%, 113/2,396, 27 states, *p *= 0.31) (Table 1, Figure 1). None of the samples from 64 cattle, 80 horses, 41 goats, or 15 pigs tested positive.

### Age, but not sex and breed, as a risk factor

**Figure 2.**
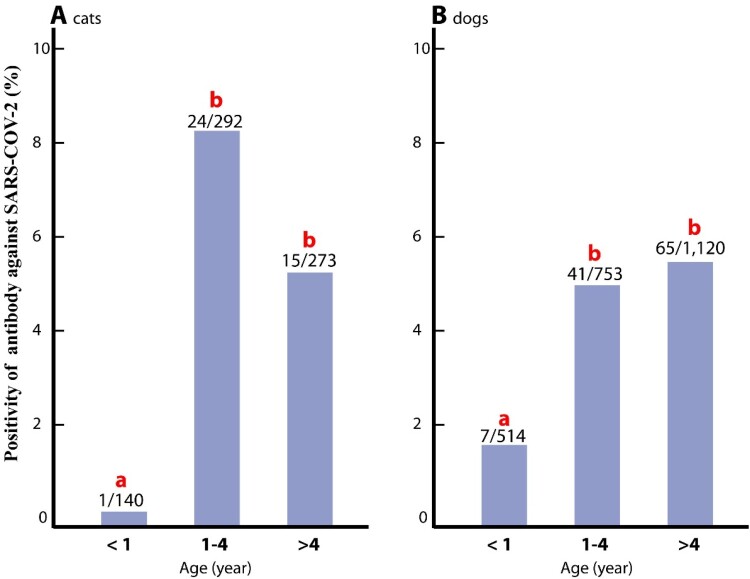
Younger cats and dogs showed significantly lower SARS-CoV-2 seroprevalence rates. The bar diagram represents three age groups (<1 year, 1–4 years, and >4 years) plotted against SARS-CoV-2 seropositivity. **(A)** In cats, the <1 year age group showed significantly lower seropositivity (0.71%) compared to the 1–4 years age group (8.2%, *p* = 0.002) and the >4 years age group (5.49%, *p* = 0.017). **(B)** In dogs, the <1 year age group had significantly lower seropositivity (1.36%) compared to the 1–4 years age group (5.4%, *p* < 0.001) and the >4 years age group (5.8%, *p* < 0.001). Different letters (a, b) signify significant differences.

This study tested 706 serum samples from cats aging from four months to 21 years (mean: 4.5 years, SD: 3.8 years), and 2,396 serum samples from dogs, aged between one month to 18 years (mean: 4.99 years, SD: 3.92 years). The seropositivity rate among cats younger than 12 months old (0.71%, 1/140) was significantly lower than in cats aged 1–4 years old (8.2%, 24/292; *P* = 0.002), and those older than four years (5.5%, 15/273; *p* = 0.017) (Figure 2). Similarly, dogs younger than 12 months (0.7%, 7/514) had a significantly lower seropositivity rate compared to those between 1–4 years of age (8.2%, 41/753; *p* < 0.001) and those older than four years (5.5%, 65/1,120; *p* < 0.001) (Figure 2).

No significant difference in SARS-CoV-2 antibody seropositivity was observed between male and female cats (22/373, 5.9% vs. 18/333, 5.4%), or between male and female dogs (61/1,193, 5.1% vs. 52/1,199, 4.3%).

In this study, the submitted samples represented 35 different feline breeds. Breeds with more than 10 submissions were Domestic Shorthair (n = 352), Ragdoll (n = 53), British Shorthair (n = 48), Domestic Longhair (n = 40), Domestic Medium Hair (n = 34), Siamese (n = 20), Devon Rex (n = 13), Persian (n = 13), Maine Coon (n = 10), and mixed breed (n = 59). The submitted canine samples represented 167 different breeds, and the breeds with more than 50 submissions included Chihuahua (n = 171), Shih Tzu (n = 121), Poodle (n = 107), Maltese (n = 88), Yorkshire Terrier (n = 86), Golden Retriever (n = 73), French Bulldog (n = 72), German Shepherd (n = 64), Labrador Retriever (n = 62), Schnauzer (n = 60), Lab Mix (n = 51) and mixed breed (n = 316) (Supplementary Files 1 & 2). There was no significant difference in seropositivity among the 35 feline breeds and 167 canine breeds included in this study.

### Feline and canine samples showed seropositivity for multiple SARS-CoV-2 variants and sublineages

The 153 feline and canine samples that tested positive using the standard sVNT were further analyzed using variant-specific sVNT kits targeting the Delta variant and five Omicron sublineages: BA.1, BA.2, BA.4/BA.5, XBB, and XBB1.5.

**Figure 3.**
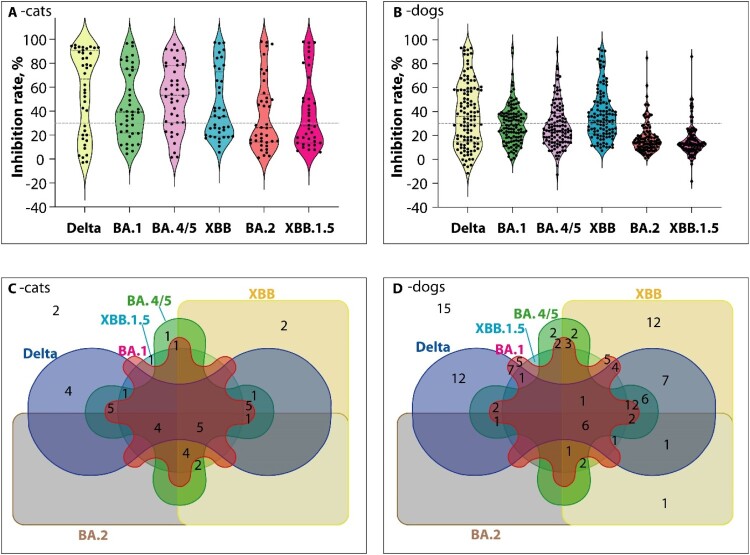
Distribution of seropositivity against SARS-CoV-2 Delta and five Omicron sublineages. The standard sVNTs identified 40 feline and 113 canine samples as SARS-CoV-2 antibody positive. These positive samples were further analyzed using variant-specific sVNT kits, with the results depicted in Violin plots (panels A and B) and Venn Diagrams (panels C and D). **A and B:** Violin plots show the inhibition rates across Delta and five Omicron sublineages in cat (A) and dog (B) samples. The horizontal dashed line indicates the ≥30% inhibition threshold for positivity. Black dots represent the distribution of inhibition rates of the individual sample. **C and D:** The interactions between variations of the seroprevalence of Delta and five Omicron sublineages across feline (C) and canine (D) samples were illustrated using a Venn diagram, which graphically depicts the intersections and distinctive features of the studied variants, enabling a comprehensive and succinct comparison. Each shape with a distinct colour represents a variant, with overlapping areas indicating the presence of multiple variants within the same samples. The numbers in the overlapping areas correspond to the count of samples that harbour each specific combination of variants.

Among the 40 feline samples that tested positive using the standard sVNT, 67.5% (27/40) were positive for the Delta variant. The positivity rates for the Omicron sublineages were as follows: 62.5% (25/40) for BA.1, 52.5% (21/40) for BA.2, 77.5% (31/40) for BA.4/BA.5, 42.5% (17/40) for XBB, and 45.0% (18/40) for XBB.1.5 (Figure 3). Five of the feline-positive samples (n = 40) tested positive for all variants and sublineages, while two tested negative for all variants. Eight feline samples tested positive for only one of the variants and sublineages, including Delta (n = 4), BA.2 (n = 1), BA.4/BA.5 (n = 2), and XBB.1.5 (n = 1) (Supplementary Files 3 & 4). The temporal distribution of seropositive cats and dogs for Delta variant and five Omicron lineages shows no significant differences in seropositivity from January to December (Supplementary Files 5 & 6).

In the 113 canine samples that tested positive using the standard sVNT, the positivity rates were as follows: 55.8% (63/113) for Delta, 46.0% (52/113) for BA.1, 58.4% (66/113) for BA.2, 37.2% (42/113) for BA.4/BA.5, 13.3% (15/113) for XBB, and 9.7% (11/113) for XBB.1.5 (Figure 3). Among the canine samples (n = 113), seven tested positive for all variants and sublineages, while 15 samples tested negative for all variants. Twelve canine samples tested positive for only one of the variants and sublineages, including Delta (n = 12), BA.1 (n = 5), BA.2 (n = 2), BA.4/BA.5 (n = 12), and XBB.1.5 (n = 1) (Supplementary Files 3 & 4).

## Discussions

This nationwide study, the largest serosurvey of its kind to date worldwide, involved samples from 3,102 apparently healthy companion animals in the USA. The research provides new insights into the risk factors associated with SARS-CoV-2, revealing higher seroprevalence rates in both cats and dogs; and offers a comprehensive comparison of seropositivity across various SARS-CoV-2 variants.

In this study, the standard sVNT revealed overall seropositivity against SARS-CoV-2 in 4.93% of the submitted samples in the USA collected in 2023. Similar seroprevalence rates were reported in cats and dogs by other studies [25,26]. However, a large-scale serosurvey conducted in 2020 [24] revealed much lower seropositivity rates in cats (0.4%) and dogs (0.0%) (Table 2, Table 3).

**Table 2.** Prevalence of SARS-CoV-2 in cats in the USA.

State	Sample size	Sampling period	Sample detection method	Seroprevalence; SARS-CoV-2 identified	Household infection status	References
48 states	956	2020	Double antigen sandwich ELISA; sVNT; VNT	0.42%	Not available	[27]
UT, WI	19	2020	VNT	21.05%	Available	[28]
MN	239	2020	N-specific ELISA; RBD-specific ELISA; VNT	7.95%; 3.00%	Not available	[21]
TX	16	2020	VNT	43.8%: clades G (*n* = 1), GH (*n* = 2), and GR (*n* = 1)	Available	[29]
AL, GA, NC	54	2020	VNT	0.00%	Available	[30]
33 states	109	2020–2021	ELISA, VNT	100%: Alpha (B.1.1.7); Delta (B.1.617.2); Epsilon (B.1.429) and lota (B.1.526)	Available	[31]
NC	21	2020–2021	ELISA, VNT	28.6%	Available	[32]
MN	204	2020–2021	ELISA	18.00%	Available	[33]
PA		2021–2022	Lateral Flow Assay, VNT	12.86%, pre-Omicron and Omicron variants	Not available	[34]
NY	79	2020–2021	Fluorescent bead-based multiplex assay, VNT	8.86%	Available	[35]
IL	1,715	2021–2023	Blocking ELISA; Lumit™ Dx SARS-CoV-2 Immunoassay, VNT	14.23%: SARS-CoV-2 variant D614G and Omicron	Not available	[36]
39 states	706	2023	sVNT	5.67%	Not available	This study

**Table 3.** Prevalence of SARS-CoV-2 in dogs in the USA.

State	Sample size	Sampling period	Sample detection method	Seroprevalence; SARS-CoV-2 identified	Household infection status	References
48 states	1,336	2020	Double antigen sandwich ELISA; sVNT; VNT	0.0%	Not available	[27]
UT and WI	37	2020	VNT	10.81%	Available	[28]
MN	510	2020	N-specific ELISA; RBD-specific ELISA; VNT	1.00%; 0.00%	Not available	[21]
TX	59	2020	VNT	11.90%: clades G (*n *= 1), GH (*n *= 2), and GR (*n *= 1)	Available	[29]
AL, GA, NC	42	2020	VNT	2.38%	Available	[30]
33 states	95	2020–2021	ELISA, VNT	100%: Alpha (B.1.1.7); Delta (B.1.617.2); Epsilon (B.1.429) and lota (B.1.526)	Available	[31]
NC	43	2020–2021	ELISA	37.5%	Available	[32]
MN	198	2020–2021	ELISA	11.60%	Available	[33]
46 states	2,396	2023	sVNT	4.72%	Not available	This study

No seropositivity was detected in samples from 14 out of 49 states in this study (Figure 1). The negativity may be attributed to the relatively small sample size submitted from these states, ranging from 3 to 29 submissions.

In this study, information on the COVID-19 status of the households where the cats and dogs reside was not available. In households with confirmed COVID-19 cases, the reported seroprevalence was found as high as 43.8% of cats [37] and 37.5% of dogs [32] (Table 2, Table 3). The varying range of reported prevalence rates in cats and dogs may be attributed to the diversity in methodologies, along with sample sizes, specific date ranges, and limited geographic scope in the USA [21,30,38]. In contrast, this study's broader geographic coverage and large sample size likely provided a more comprehensive view.

In this study, no significant differences were observed in the seropositive rates between cats (5.7%) and dogs (4.7%), a finding supported by other reports [39,40]. However, other studies on SARS-CoV-2 in cats across different regions of the USA, with sample sizes ranging from 16 to 1,715, have shown varying seroprevalence rates from 0% to 43.8% in cats [30,37,41] (Table 2) and 0% to 37.5% in dogs [21,24,32,38] (Table 3). Studies with high prevalences often targeted animals in homes with known SARS-CoV-2 infections [29,31].

Understanding variables such as age, sex, and breed is crucial for characterizing the epidemiology of SARS-CoV-2 in cats and dogs. This study found that younger animals (<1 year old) exhibited significantly lower seropositivity rates compared to older ones. This finding is consistent with other reports that also found a positive association between older age and higher seropositivity in dogs [40,42]; However, age was not associated with seropositivity in cats in some studies [40], whereas other research indicated that young cats had higher seropositivity than older cats [43]. In cats and dogs under 1 year of age, the lower seropositivity could be attributed to their shorter exposure time to SARS-CoV-2 compared to older age groups, leading to lower rates of detectable antibodies. This age-related disparity emphasizes the importance of considering age when assessing the risk and extent of SARS-CoV-2 exposure in companion animals.

Regarding sex and breed, no significant differences were observed in SARS-CoV-2 antibody seropositivity between males and females or among 35 feline breeds and 167 canine breeds, aligning with previous studies [43–45]. However, some studies have reported that purebred and male cats exhibit higher levels of seroprevalence [26].

In this study, six sVNTs were used to measure the neutralizing activity of antibodies against SARS-CoV-2 Delta and Omicron variants (BA.1, BA.2, BA.4/BA.5, XBB, and XBB.1.5) in seropositive cats and dogs. A surprising finding was that a significant number of companion cats (30/40, 75.0%) and dogs (67/113, 59.3%) tested positive for at least two among the Delta variant and five Omicron sublineages. Research by Kimmerlein et al. [40] suggests that antibodies persisted for up to 650 days in cats and 828 days in dogs. This suggests that companion animals might retain neutralizing antibodies to SARS-CoV-2 for a more extended period than initially thought. In addition, 17 positive samples determined by standard ELISA were found negative for any tested variants of the SARS-Cov-2 antibodies in this study. These samples might carry antibodies against other variants not covered by the six sVNT kits used in this study.

The samples in this study were collected in 2023, after the emergence of Omicron XBB and XBB.1.5. Interestingly, the study observed a relatively lower percentage of seropositivity for XBB and XBB.1.5 in both cats and dogs compared to Delta and other Omicron sublineages, with this difference being particularly notable in canine samples. This could be attributed to the superior immune escape capabilities of XBB and XBB.1.5 compared to previous Omicron sublineages reported in humans [46–48].

In agreement with the detection of antibodies against multiple variants identified by FDA-approved specific sVNT kits in this study, other studies used PCR and sequencing to identify Alpha (B.1.1.7), Delta (B.1.617.2), Epsilon (B.1.429), lota (B.1.526), and Omicron variants in cats and dogs [31] [36]. The specific sVNT tests used in this study offer convenience in terms of sample handling, equipment requirements, cost, and technical complexity, making it an attractive alternative to molecular approaches for the monitoring of the COVID-19 epidemiology. Although FDA-approved sVNT kits have demonstrated high specificity in detecting antibodies against multiple variants, potential cross-reactivity and antibody imprinting in cats and dogs warrant further investigation, and the specificity of these kits should be validated further.

Both humans and companion animals are susceptible to the virus, and the disease may present in an asymptomatic or subclinical manner, further elevating the risk of transmission. The seroprevalence rate of 4.93% among cats and dogs in this large cohort study is significant, and antibodies from SARS-CoV-2 infections in companion animals are likely to persist for a long time. Given the large and nationwide population of companion animals, their close interaction with humans, and the ability of SARS-CoV-2 to replicate and be transmitted between humans and animals, companion animals could serve as valuable sentinels for monitoring the spread of SARS-CoV-2 and detecting emerging variants.

The seroprevalence of SARS-CoV-2 in companion cats and dogs, while less than 5% in this 2023 study, can still provide valuable insights into tracking COVID-19. In humans, a study conducted from December 2019 to December 2021 reported an overall 9% seroprevalence in the USA [49], but by January – March 2022, 93.5% of people aged ≥16 years had antibodies from previous infection or vaccination [50]. Of these, 39% had antibodies from vaccination alone, 20.5% from infection alone, and 34.1% from both [50]. This sharp rise in human seropositivity is largely due to widespread vaccination, making it difficult to distinguish between natural infection and vaccine-induced immunity. In contrast, animals provide a more direct measure of natural infection since they are not vaccinated against SARS-CoV-2.

Additionally, the significant decline in human COVID-19 molecular testing has limited our ability to track circulating variants. This study addresses that gap by employing a specific ELISA to detect antibodies against different SARS-CoV-2 variants in animals. Interestingly, antibody-positive cases against all five SARS-CoV-2 Omicron lineages were identified in January, the first month of sample collection in this study (Table 1). Notably, three of these four Omicron sublineages were first reported in humans in the USA by December 2022. The timely detection of specific anti-SAR-CoV-2 antibodies in cats and dogs further highlights the critical role of companion animals in monitoring COVID-19 epidemiology. Serosurveillance in companion animals can therefore complement declining human testing and serve as a sentinel system for detecting variants, providing valuable epidemiological insights.

In tracking COVID-19, each of the surveillance tools such as seroprevalence studies in companion animals and molecular testing in wastewater offer unique and complementary insights. Seroprevalence in dogs and cats provides valuable data on animal exposure to SARS-CoV-2, which may reflect the viral circulation in human populations closely interacting with these pets. This tool can reveal historical patterns of infection and immune response over time, especially as antibody levels persist after active infection.

On the other hand, wastewater surveillance detects viral RNA shed by infected individuals in the community, often capturing asymptomatic or undiagnosed cases [51–55]. This method provides real-time insights into ongoing viral transmission, offering early warnings of outbreaks. Wastewater analysis is not limited by testing individuals, making it cost-effective for large-scale monitoring, while seroprevalence offers detailed information on the extent of past infections in specific populations. Both tools have limitations. Seroprevalence is retrospective, focusing on past infections, while wastewater monitoring lacks specificity regarding which individuals or species are infected. However, when used together, they complement one another by providing both real-time and historical data on virus spread, helping to build a comprehensive picture of COVID-19 dynamics in human and animal populations.

In conclusion, this study provides critical and updated insights into the seroprevalence of SARS-CoV-2 variants among companion animals in the United States. A significant portion of cats and dogs have been exposed to multiple variants, including Delta and five Omicron sublineages. The findings underscore the role of companion animals as potential sentinels in the epidemiology of COVID-19. The age-related differences in seropositivity highlight the need for ongoing surveillance to better understand the dynamics of SARS-CoV-2 transmission in these populations. These results emphasize the importance of continued monitoring and research to inform public health strategies and mitigate the risks of zoonotic disease transmission.

## Supplementary Material

Supplementary Files.docx
