# Supplementary material for: Nationwide seroprevalence of SARS-CoV-2 Delta variant and five Omicron sublineages in companion cats and dogs in the USA: insights into their role in COVID-19 epidemiology
Source: Emerg Microbes Infect. 2024 Dec 5;14(1):2437246. doi: 10.1080/22221751.2024.2437246 (PMC11636146; doi:10.1080/22221751.2024.2437246)
Supplement: Supplementary Files.docx [file TEMI_A_2437246_SM0452.docx]

**Supplementary File 1: Summary of demographics for SARS-CoV-2 seropositive cats in this study**

| **Sl. no.** | **Date** | **Age (year)** | **Breed** | **Sex** | **State** |
| --- | --- | --- | --- | --- | --- |
| 1 | 1/13/2023 | 4.5 | Domestic Shorthair | Male | OH |
| 2 | 1/18/2023 | 2.0 | Domestic Shorthair | Male | NY |
| 3 | 1/18/2023 | 2.0 | Domestic Shorthair | Male | NY |
| 4 | 1/19/2023 | 4.0 | Mixed Breed | Female | Not available |
| 5 | 1/24/2023 | 3.0 | British Shorthair | Male | NY |
| 6 | 1/24/2023 | 2.0 | Domestic Shorthair | Male | NY |
| 7 | 1/31/2023 | 3.0 | Ragdoll | Female | NY |
| 8 | 2/10/2023 | 3.0 | Domestic Shorthair | Male | WA |
| 9 | 3/2/2023 | 3.0 | Ragdoll | Male | Not available |
| 10 | 3/16/2023 | 7.0 | Domestic Shorthair | Male | PA |
| 11 | 3/27/2023 | 3.0 | Domestic Shorthair | Male | Not available |
| 12 | 4/10/2023 | 2.0 | Domestic Shorthair | Male | VA |
| 13 | 4/13/2023 | 4.0 | Domestic Shorthair | Male | NY |
| 14 | 4/20/2023 | 6.9 | Siamese | Male | Not available |
| 15 | 4/21/2023 | 3.0 | Domestic Shorthair | Female | GA |
| 16 | 4/24/2023 | 2.0 | Domestic Shorthair | Male | AL |
| 17 | 5/9/2023 | 1.5 | British Longhair | Male | PA |
| 18 | 5/10/2023 | 2.0 | Domestic Shorthair | Female | MI |
| 19 | 5/11/2023 | 11.0 | Domestic Longhair | Female | CA |
| 20 | 6/6/2023 | 5.0 | British Shorthair | Male | IL |
| 21 | 6/8/2023 | 3.8 | Domestic Shorthair | Female | WA |
| 22 | 6/22/2023 | 5.0 | Domestic Shorthair | Female | WI |
| 23 | 7/6/2023 | 2.9 | Domestic Medium Hair | Female | NV |
| 24 | 7/6/2023 | 4.0 | Domestic Shorthair | Male | NV |
| 25 | 7/11/2023 | 4.2 | Ragdoll | Female | Not available |
| 26 | 8/22/2023 | 5.0 | Scottish Fold Mixed | Female | CA |
| 27 | 9/13/2023 | 6.0 | Domestic Shorthair | Female | GA |
| 28 | 9/13/2023 | 5.0 | Domestic Shorthair | Male | GA |
| 29 | 9/15/2023 | 5.0 | Mixed Breed | Female | Not available |
| 30 | 9/25/2023 | 3.0 | Domestic Longhair | Male | AL |
| 31 | 10/6/2023 | 8.0 | Domestic Shorthair | Female | SC |
| 32 | 10/24/2023 | 2.0 | Domestic Longhair | Female | Not available |
| 33 | 10/24/2023 | 2.0 | Domestic Longhair | Male | Not available |
| 34 | 10/31/2023 | 5.0 | Domestic Shorthair | Male | NY |
| 35 | 11/17/2023 | 1.0 | Domestic Shorthair | Female | Not available |
| 36 | 11/17/2023 | 2.0 | Domestic Shorthair | Male | Not available |
| 37 | 12/4/2023 | 6.0 | Mixed Breed | Male | Not available |
| 38 | 12/8/2023 | 13.0 | Domestic Shorthair | Female | MA |
| 39 | 12/20/2023 | 2.0 | Mixed Breed | Female | Not available |
| 40 | 12/22/2023 | 4.0 | Ragdoll | Female | MN |

**Supplementary File 2: Summary of demographics for SARS-CoV-2 seropositive dogs in this study**

| **Sl. no.** | **Date** | **Age (year)** | **Breed** | **Sex** | **State** |
| --- | --- | --- | --- | --- | --- |
| 1 | 1/12/2023 | 9.0 | Mixed Breed | Male | MS |
| 2 | 1/20/2023 | 3.0 | Jack Russell Terrier | Male | Not available |
| 3 | 1/20/2023 | 5.0 | Mixed Breed | Male | Not available |
| 4 | 1/25/2023 | 6.0 | Poodle Mix | Male | HI |
| 5 | 2/3/2023 | 4.0 | Labrador Retriever | Female | AR |
| 6 | 2/10/2023 | 12.0 | Labrador Retriever | Male | MT |
| 7 | 2/13/2023 | 14.0 | Shepherd Mix | Male | KY |
| 8 | 2/17/2023 | 3.0 | German Shepherd | Female | TX |
| 9 | 2/17/2023 | 2.3 | Mixed Breed | Male | Not available |
| 10 | 2/22/2023 | 2.0 | Yorkshire Terrier | Female | AL |
| 11 | 2/23/2023 | 9.0 | Mixed Breed | Male | FL |
| 12 | 2/23/2023 | 6.0 | Schnauzer | Male | Not available |
| 13 | 2/24/2023 | 7.0 | Labrador Retriever | Male | FL |
| 14 | 2/24/2023 | 12.0 | Belgian Malinois | Female | FL |
| 15 | 3/1/2023 | 4.0 | Chihuahua | Male | FL |
| 16 | 3/7/2023 | 2.0 | Siberian Husky | Male | NY |
| 17 | 3/8/2023 | 5.0 | French Bulldog | Male | Not available |
| 18 | 3/10/2023 | 3.0 | Mastiff Mix | Male | ID |
| 19 | 3/10/2023 | 2.0 | Golden Retriever | Female | Not available |
| 20 | 3/10/2023 | 8.0 | Doberman Pischer | Male | Not available |
| 21 | 3/10/2023 | 0.7 | Yorkshire Terrier | Male | Not available |
| 22 | 3/13/2023 | 4.0 | Mixed Breed | Male | Not available |
| 23 | 3/13/2023 | 8.0 | Mixed Breed | Female | Not available |
| 24 | 3/14/2023 | 5.0 | Boykin Spaniel | Female | MI |
| 25 | 3/16/2023 | 3.0 | Labrador Retriever | Male | HI |
| 26 | 3/17/2023 | 7.0 | Weimaraner | Male | UT |
| 27 | 3/17/2023 | 2.0 | Pit Bull | Female | HI |
| 28 | 3/20/2023 | 3.0 | Pit Bull | Male | OH |
| 29 | 3/24/2023 | 2.0 | Rottweiler Mix | Male | Not available |
| 30 | 4/3/2023 | 9.0 | Mixed Breed | Female | Not available |
| 31 | 4/4/2023 | 1.0 | Shiba Inu | Male | CA |
| 32 | 4/4/2023 | 7.0 | Lab Mix | Female | GA |
| 33 | 4/6/2023 | 2.0 | Lab Mx | Female | HI |
| 34 | 4/7/2023 | 0.4 | Boerboel | Male | LA |
| 35 | 4/10/2023 | 0.4 | Mixed Breed | Female | Not available |
| 36 | 4/11/2023 | 6.0 | Pekingese | Male | FL |
| 37 | 4/11/2023 | 4.0 | Mixed Breed | Female | FL |
| 38 | 4/11/2023 | 5.0 | Pit Bull Mix | Female | VA |
| 39 | 4/12/2023 | 6.0 | Chihuahua | Male | Not available |
| 40 | 4/24/2023 | 1.0 | Mixed Breed | Male | FL |
| 41 | 5/3/2023 | 10.0 | Dachshund | Female | AL |
| 42 | 5/4/2023 | 2.0 | Lab Mix | Female | TN |
| 43 | 5/4/2023 | 3.0 | Bulldog | Male | WV |
| 44 | 5/9/2023 | 5.0 | Chihuahua Mix | Female | KY |
| 45 | 5/9/2023 | 5.0 | Husky Mix | Female | GA |
| 46 | 5/9/2023 | 14.0 | Cocker Spaniel | Male | PA |
| 47 | 5/19/2023 | 2.1 | Shih Tzu | Female | NY |
| 48 | 5/19/2023 | 7.0 | German Shepherd | Male | FL |
| 49 | 5/22/2023 | 5.0 | Poodle Mix | Male | CA |
| 50 | 5/22/2023 | 13.0 | Portuguese Water Dog | Female | GA |
| 51 | 5/24/2023 | 4.0 | Belgian Malinois | Male | TN |
| 52 | 5/25/2023 | 12.0 | Chihuahua | Male | PA |
| 53 | 5/25/2023 | 4.0 | Schnauzer | Female | AL |
| 54 | 6/2/2023 | 5.0 | Bernese Mountain Dog | Male | Not available |
| 55 | 6/2/2023 | 14.0 | Shepherd Mix | Male | KY |
| 56 | 6/2/2023 | 5.0 | Lab Mix | Male | AL |
| 57 | 6/5/2023 | 5.0 | Belgian Malinois | Female | MI |
| 58 | 6/7/2023 | 9.0 | Lab Mix | Male | MS |
| 59 | 6/8/2023 | 5.0 | Mixed Breed | Male | Not available |
| 60 | 6/8/2023 | 8.0 | Golden Retriever Mix | Male | PA |
| 61 | 6/9/2023 | 4.0 | French Bulldog | Female | NV |
| 62 | 6/15/2023 | 5.9 | Pit Bull | Female | FL |
| 63 | 6/15/2023 | 4.0 | Alaskan Malamute | Female | MI |
| 64 | 6/28/2023 | 7.0 | Miniature Schnauzer | Male | LA |
| 65 | 6/28/2023 | 10.0 | Mixed Breed | Female | PA |
| 66 | 7/10/2023 | 7.0 | Shepherd Mix | Female | TX |
| 67 | 7/11/2023 | 8.0 | Maltese | Female | CA |
| 68 | 7/14/2023 | 7.0 | Schnauzer | Male | DE |
| 69 | 7/18/2023 | 6.0 | Goldendoodle | Male | AL |
| 70 | 7/26/2023 | 4.0 | Rottweiler | Male | FL |
| 71 | 7/26/2023 | 8.0 | French Bulldog | Female | FL |
| 72 | 7/31/2023 | 2.5 | Mixed Breed | Male | Not available |
| 73 | 8/4/2023 | 4.0 | Cocker Spaniel | Male | Not available |
| 74 | 8/4/2023 | 4.0 | Doberman Pinscher | Female | AL |
| 75 | 8/8/2023 | 12.0 | American Bully | Male | CO |
| 76 | 8/9/2023 | 4.0 | Pyrenees Mix | Female | MT |
| 77 | 8/14/2023 | 5.7 | Mixed Breed | Female | OK |
| 78 | 8/15/2023 | 4.0 | Mixed Breed | Male | OH |
| 79 | 8/15/2023 | 0.6 | Pekingese | Female | FL |
| 80 | 08/22/2023 | 4.0 | Pit Bull | Female | PA |
| 81 | 08/22/2023 | 0.3 | Whippet | Female | PA |
| 82 | 8/22/2023 | 5.0 | German Shepherd | Female | GA |
| 83 | 8/22/2023 | 7.0 | Poodle Mix | Female | KY |
| 84 | 8/28/2023 | 2.0 | Cocker Spaniel | Male | NC |
| 85 | 8/29/2023 | 4.0 | Lab Mix | Female | GA |
| 86 | 8/29/2023 | 4.0 | Lab Mix | Male | GA |
| 87 | 8/29/2023 | 9.0 | Yorkshire Terrier | Female | LA |
| 88 | 9/7/2023 | 11.0 | Dachshund | Male | PA |
| 89 | 9/22/2023 | 7.0 | Yorkshire Terrier | Female | MA |
| 90 | 9/22/2023 | 4.0 | Schnauzer | Female | FL |
| 91 | 9/22/2023 | 11.0 | Mixed Breed | Male | FL |
| 92 | 10/5/2023 | 15.0 | Border Collie | Female | PA |
| 93 | 10/6/2023 | 5.0 | Mixed Breed | Female | Not available |
| 94 | 10/13/2023 | 6.0 | Great Dane | Male | FL |
| 95 | 10/18/2023 | 7.0 | Mixed Breed | Female | Not available |
| 96 | 10/27/2023 | 4.0 | Boxer Mix | Male | Not available |
| 97 | 11/02/2023 | 12.0 | Maltese | Male | TN |
| 98 | 11/02/2023 | 9.0 | Shih Tzu Mix | Male | MO |
| 99 | 11/10/2023 | 6.0 | Schnauzer | Female | Not available |
| 100 | 11/14/2023 | 5.0 | Dalmatian | Male | CA |
| 101 | 11/17/2023 | 4.0 | Rat Terrier | Female | VA |
| 102 | 11/21/2023 | 4.0 | Doberman Pinscher | Male | TN |
| 103 | 12/7/2023 | 4.0 | Labrador Retriever | Male | PA |
| 104 | 12/8/2023 | 12.0 | American Staffordshire Terrier | Female | GA |
| 105 | 12/15/2023 | 2.5 | Doodle | Female | GA |
| 106 | 12/15/2023 | 8.0 | Chihuahua | Female | MI |
| 107 | 12/18/2023 | 5.0 | Cocker Spaniel | Male | Not available |
| 108 | 12/20/2023 | 4.0 | Poodle Mix | Female | MA |
| 109 | 12/20/2023 | 3.0 | Mixed Breed | Female | Not available |
| 110 | 12/20/2023 | 10.0 | Poodle | Female | Not available |
| 111 | 12/29/2023 | 5.0 | Goldendoodle | Male | TN |
| 112 | 12/29/2023 | 7.0 | Husky | Male | PA |
| 113 | 12/29/2023 | 5.0 | Siberian Husky | Male | PA |

**
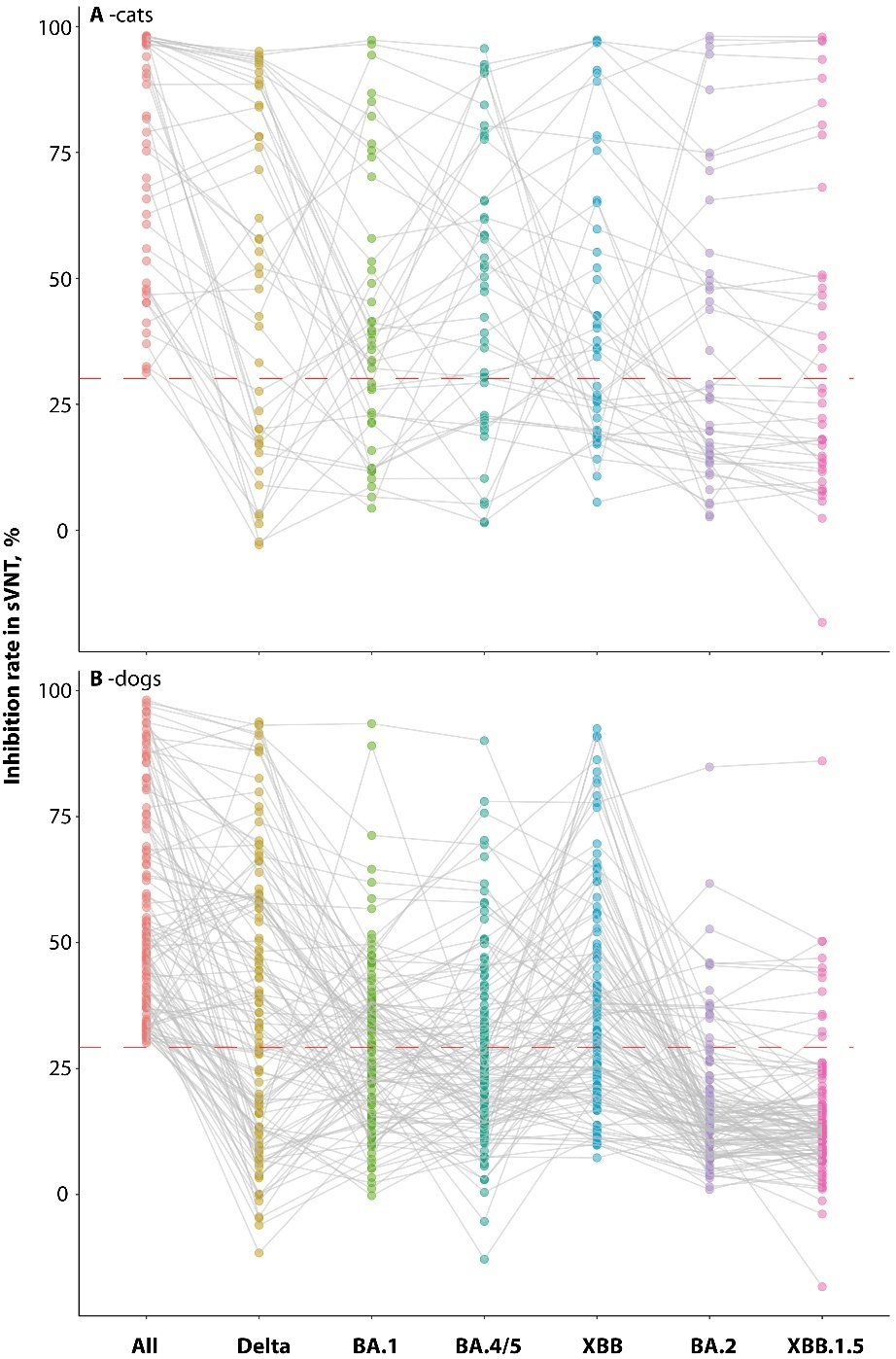
**

**Supplementary File 3. Antibody-positive samples against SARS-CoV-2 determined by regular sVNT, and specific sVNTs against Delta variant and five Omicron sublineages**. The standard sVNTs identified 40 feline and 113 canine samples as SARS-CoV-2 antibody positive. These positive samples were further analyzed using variant-specific sVNT kits, with the results depicted in cats (panel A) and dogs (panel B). The RStudio© software (Version 2023.12.1+402) was used to draw the figure, and the same sample was connected with the line.

**
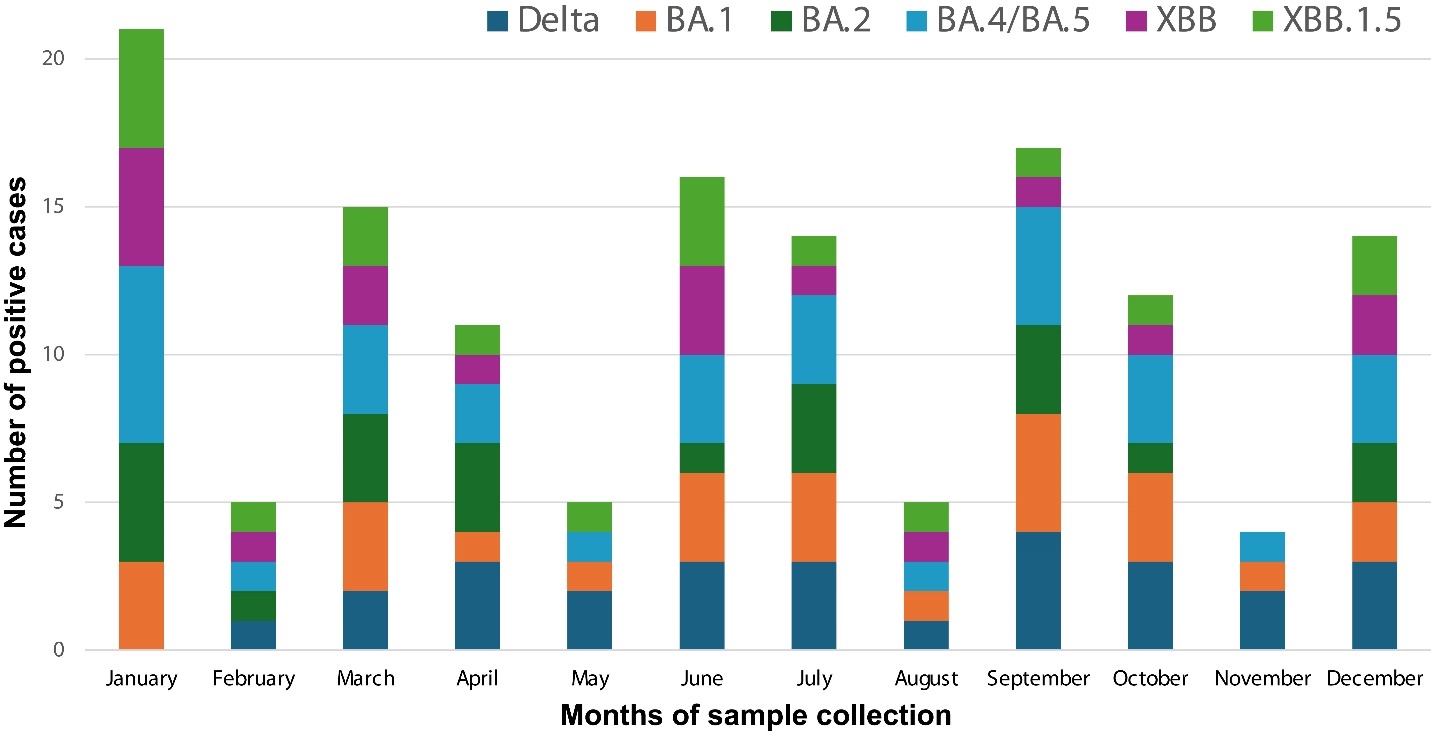
**

**Supplementary File 4. Temporal distribution of seropositive cats in this study.** Six SARS-CoV-2 variants/sublineages are shown in different colors, and the number corresponding to the Y-axis indicates the case numbers, being seropositive for the designated variant/sublineage in different months (X-axis). No seasonal prevalence was observed in seropositivity between different months.

**
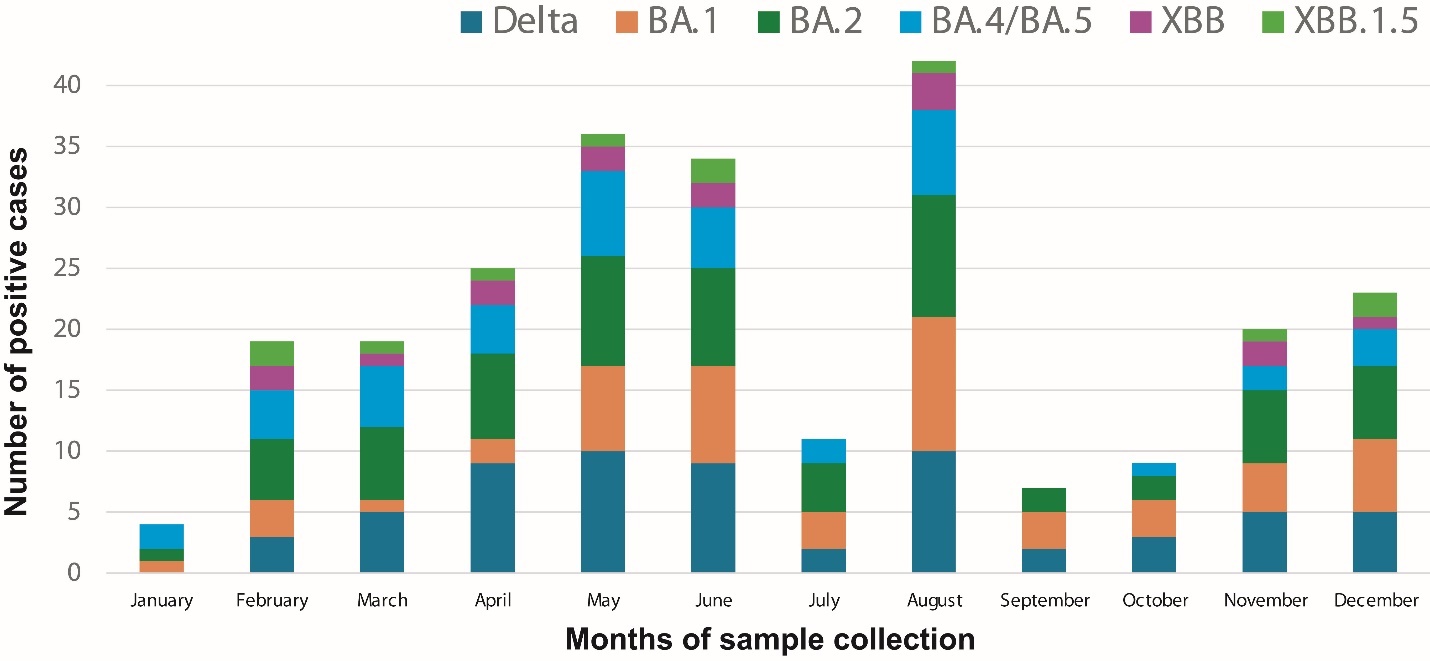
**

**Supplementary File 5. Temporal distribution of seropositive dogs in this study.** Six SARS-CoV-2 variants/sublineages are shown in different colors, and the number corresponding to the Y-axis indicates the case numbers, being seropositive for the designated variant/sublineage in different months (X-axis). The sero-positivity tended to be lower in January, February, July, September and October but no significant difference in seropositivity between months was identified.

**Supplementary File 6: Temporal distribution of seropositive dogs in this study**

|  | **Delta** | **BA.1** | **BA.2** | **BA.4/BA.5** | **XBB** | **XBB.1.5** |
| --- | --- | --- | --- | --- | --- | --- |
| **January** | 0* | 1 | 1 | 2 | 0 | 0 |
| **February** | 3 | 3 | 5 | 4 | 2 | 2 |
| **March** | 5 | 1 | 6 | 5 | 1 | 1 |
| **April** | 9 | 2 | 7 | 4 | 2 | 1 |
| **May** | 10 | 7 | 9 | 7 | 2 | 1 |
| **June** | 9 | 8 | 8 | 5 | 2 | 2 |
| **July** | 2 | 3 | 4 | 2 | 0 | 0 |
| **August** | 10 | 11 | 10 | 7 | 3 | 1 |
| **September** | 2 | 3 | 2 | 0 | 0 | 0 |
| **October** | 3 | 3 | 2 | 1 | 0 | 0 |
| **November** | 5 | 4 | 6 | 2 | 2 | 1 |
| **December** | 5 | 6 | 6 | 3 | 1 | 2 |
| **Total** | **63** | **52** | **66** | **42** | **15** | **11** |

* indicates the number of cases, being seropositive for the designated variant/sublineage.
